# A functional variant in promoter region of platelet-derived growth factor-D is probably associated with intracerebral hemorrhage

**DOI:** 10.1186/1742-2094-9-26

**Published:** 2012-01-30

**Authors:** Yongyi Bai, Jingzhou Chen, Kai Sun, Yibo Wang, Rutai Hui

**Affiliations:** 1 State Key Laboratory of Cardiovascular Disease, Sino-German Laboratory for Molecular Medicine and Key Laboratory for Clinical Cardiovascular Genetics, Fuwai Hospital, National Center for Cardiovascular Diseases, Chinese Academy of Medical Sciences and Peking Union Medical College, Beijing, 100037, People's Republic of China; 2 Department of Geriatric Cardiology, Chinese PLA General Hospital, Beijing 100853. People's Republic of China

**Keywords:** Platelet-derived growth factor D, genetics, intracerebral hemorrhage, risk factors

## Abstract

**Background:**

Platelet-derived growth factor D (PDGF-D) plays an important role in angiogenesis, vessel remodeling, inflammation and repair in response to injury. We hypothesized that genetic variation in *PDGFD *gene might alter the susceptibility to stroke.

**Findings:**

We determined the genotypes of a single nucleotide polymorphism (SNP) (-858A/C, rs3809021) in 1484 patients with stroke (654 cerebral thrombosis, 419 lacunar infarction, 411 intracerebral hemorrhage [ICH]) and 1528 control subjects from an unrelated Chinese Han population and followed the stroke patients up for a median of 4.5 years.

The -858AA genotype showed significantly increased risk of ICH (dominant model: odds ratio [OR] 1.29, 95% confidence interval [CI] 1.00-1.68, *P *= 0.05; additive model: OR 1.24, 95% CI 1.01-1.52, *P *= 0.04) than wild-type genotype. Further analyses showed that -858AA genotype conferred about 2-fold increase in risk of non-hypertensive ICH (dominant model: OR 2.1, 95%CI 1.34-3.29, *P *= 0.001; additive model: OR 1.75, 95% CI 1.24-2.46, *P *= 0.001). After a median follow-up of 4.5 years, -858AA genotype was associated with a reduced risk of ICH recurrence (dominant model: adjusted hazard ratio [HR] 0.09, 95%CI 0.01-0.74, P = 0.025; additive model: HR 0.21, 95% CI 0.04-1.16, *P *= 0.073) in non-hypertensive patients.

**Conclusions:**

The -858AA genotype is probably associated with risk for non-hypertensive ICH. Further studies should be conducted to reveal the role of PDGF-D at various stages of ICH development--beneficial, or deleterious.

## Introduction

Stroke is a leading cause of death and disability in China [1]. The number of patients who die from stroke is more than three times that from coronary heart disease in China; in contrast coronary heart disease-related mortality is 2 to 3 times greater than the stroke-related mortality in Western nations [2,3]. Each year, 1.5 to 2 million people have a first-ever stroke, and more than one million die from stroke-related causes in China [4-6]. Among all subtypes of stroke, intracerebral hemorrhage (ICH) accounts for 10 to 15 percent of strokes in most western populations, whereas up to 55% of strokes in the Chinese population are ICH [7,8]. The reasons for the high incidence of stroke, especially the hemorrhagic subtype, among the Chinese population, remain unknown.

Hypertension is the most important cause for spontaneous, non-traumatic ICH both in western nations and in Chinese population [9,10]. However, non-hypertensive intracerebral hemorrhages, which may have different pathologies, mechanisms and genetic background from hypertensive ICH, are not rare. Given that there are limited modifiable risk factors for ICH and few proven preventative therapies other than control of hypertension, the most promising route to prevention and therapeutics may be identification of novel genetic variants with a role in ICH [11]. Identification of genetic polymorphisms associated with hypertensive and non-hypertensive ICH would facilitate risk stratification as well as illuminate the underlying biology.

Platelet-derived growth factor D (PDGF-D) is the most recently discovered member of the PDGF family and signals mainly through PDGF receptor-β (PDGFR-β) [12]. PDGF-D has been observed to stimulate expression of MMP-2 and MMP-9 in vascular smooth muscle cells (SMCs) and degradation of vascular basement membrane, which may lead to a loss of vessel wall integrity [13]. It has also been reported to be important in the processes of inflammation, angiogenesis and vessel remodeling in response to injury [14,15]. We therefore hypothesized that alternations in promoter region of PDGF-D gene might be associated with ICH. To test this hypothesis, we investigated the association of genetic variants in *PDGFD *with the risk of first-ever ICH in a large case-control stroke study in China. We also prospectively followed patients with primary ICH for a median 4.5 years and assessed the relationships of *PDGFD *variants to the risk of recurrence.

## Methods

The study was approved by both the ethics committee of FuWai Hospital and the local ethics committees of collaborative hospitals. All participants reported themselves to be of Han nationality and provided written informed consent.

### Study population

This is a multicenter study for assessment of risk factors of stroke sponsored by the Ministry of Science and Technology of China (973 Project). Initially, 2000 patients with stroke and 2000 control subjects were recruited as described previously [10,16].

Stroke was defined as a sudden onset of nonconvulsive and focal neurological deficit persisting for > 24 hours. Cerebral thrombosis was diagnosed if the patient had the characteristics of cerebral cortical dysfunction, and CT scan or MRI performed within 28 days of onset either showed an area of low attenuation in a region compatible with the clinical symptoms or failed to identify an alternative cause of the symptoms. For CT/MRI readers, the intrareader variability was not calculated. Among all subtypes of hemorrhagic stroke, only spontaneous, non-traumatic, primary ICH was in the range of selection. Other types of stroke, including transient ischemic attack, subarachnoid hemorrhage, embolic brain infarction, brain tumors, and cerebrovascular malformation, and severe systemic diseases such as collagenosis, endocrine and metabolic disease (except diabetes mellitus [DM]), inflammation, liver, neoplastic, or renal diseases were in the range of exclusion.

Before data analysis, 516 stroke cases and 472 controls were excluded for indefinite diagnosis (24 cases), absence of plasma (76 cases and 93 controls), insufficient DNA (355 cases and 309 controls), and failure of genotyping (61cases and 70controls). No significant differences were found in clinical characteristics between the included and excluded subjects. Finally, 1484 patients (654 cerebral thrombosis, 419 lacunar infarction, 411 ICH) and 1528 controls were eligible for genetic analysis.

### Follow-up and Outcome Assessment

To assess whether genetic variations contributed to the risk of ICH recurrence after the first-ever ICH, we prospectively followed this stroke population through May 31, 2006 by a standard questionnaire and telephone contact by physician investigators. During a median 4.5 years (range, 0.1 to 5.4 years) of follow-up for 411 patients with ICH, 14 participants were lost and excluded from analysis. Follow-up data were completed for 397 stroke patients (302 hypertensive and 95 non-hypertensive, follow-up rate 96.5%).

### Biochemical Variable Determination and Clinical Data Collection

Blood samples were obtained after at least a 12-hour overnight fasting period and pre-chilled vacutainers and immediately centrifuged. The biochemical variable determination and clinical data collection were performed as described previously [10,16].

### DNA isolation, genetic variant selection and genotyping

DNA was extracted from the buffy coat fraction of centrifuged blood using the QIAmp Blood Kit (Qiagen, Chatsworth, CA). The *PDGF-D *gene is located on chromosome 11q22.3 [12]. The selection of genetic variants was based on the HapMap CHB sample using the pairwise option of the Haploview version of Tagger program. A minimum r^2 ^of 0.8 was chosen as a threshold for all analyses. Only one Tagger SNP (rs3809021, -858A/C) with a minor allele frequency more than 5% was genotyped within the promoter region of *PDGFD *[17]. Sequence inspection and database searches have indicated that the single-base substitution at -858 site could affect DNA binding of several transcription factors, including Ap-1, c-Jun, c-Fos and Sp1-like factors, made this variant our prime candidate.

Detailed descriptions of genotyping were shown in **[**Additional file 1**]**.

### Luciferase reporter assays

We investigated whether the variant at position -858 affects the transcriptional activity of the *PDGFD *promoter (from -1168 to +185) in HCT-116 cells. The detailed descriptions of the luciferase reporter assays were shown in **[**Additional file 2**]**.

### Statistical Analysis

The distribution of continuous variables was tested for normality with a one-sample Kolmogorov-Smirnov test. Because the level of triglyceride was highly skewed, the Mann-Whitney nonparametric method was applied to compare the differences between patients and controls. Continuous variables were compared by unpaired Student *t *test or one-way ANOVA, and the categorical variables were compared by chi-square test.

Hardy-Weinberg equilibrium of each variant was tested using chi-square test. Odds ratios (ORs) and 95% confidence interval (CI) were used to determine the association between genotypes and the risk of ICH. Multivariate unconditional logistic regression was used to estimate OR and 95% CI after adjustment for age, gender, body mass index (BMI), hypertension, DM, HDL cholesterol (HDL-C), Non-HDL cholesterol (non-HDL-C), total cholesterol (TC), triglyceride (TG), drinking and smoking status. Multiple testing was adjusted using Bonferroni correction.

The Kaplan-Meier analysis and Cox proportional hazards models were used to describe the association of genotypes with incidence of ICH recurrence and cardiovascular deaths. Follow-up began on the date of diagnosis of the first index ICH and continued until the date of a recurrent stroke or the end of follow-up period. Hazard Ratio (HRs) and 95% CI were estimated using multivariate Cox proportional hazards models after adjustment for age, gender, BMI, hypertension, HDL-C, TC, TG, DM, smoking status, and alcohol intake.

All reported P values are 2-sided, and *P *< 0.05 is considered statistically significant. Analyses were performed with SPSS software version 11.0 (SPSS Inc, Chicago), and Stata software version 9.0 (Stata Corp, College Station, Tex). The power analysis was performed with the software Quanto (http://hydra.usc.edu/gxe/).

## Results

### Characteristics of the Subjects

Proportions from each of the clinical sites for cases and controls were shown in **[**Additional file 3**]**. The demographic and clinical characteristics of the subjects in the present study are shown in Table 1. ICH patients had a higher prevalence of conventional vascular risk factors, including higher in blood pressure, fasting blood glucose, cigarette smoking, lower in HDL-C, TC and non-HDL-C.

**Table 1:** Clinical characteristics of cases and control subjects

Characteristics	Control Subjects	Thrombosis Stroke	Lacunar Stroke	ICH
**n**	1528	654	419	411
**Age, yrs**	60.1(8.5)	61.4(9.8)**	61.1(8.3)**	58.4(9.5)**
**Male gender, %**	60.2	62.7	62.6	62.7
**BMI, kg/m^2^**	24.2(3.4)	24.4(3.6)*	24.5(3.1)*	24.1(3.6)
**HDL-C, mmol/L**	1.06(0.31)	0.88(0.26)**	0.93(0.27)**	0.90(0.31)**
**Non-HDL-C, mmol/L**	3.94(0.96)	3.97(1.02)	3.83(0.94)	3.64(0.94)**
**TC, mmol/L**	4.99(0.99)	4.86(1.04)*	4.77(0.95)**	4.55(1.00)**
**TG, mmol/L**	1.47(13.5)	1.73(8.41)**	1.72(12.9)**	1.48(13.7)
**Smoking, %**	38.4	45.6*	46.7*	47.9*
**Drinking, %**	32.5	40.2**	41.7**	42.1**
**Hypertension history, %**	28.9	63.6**	64.1**	76.3**
**DM history, %**	6.2	16.4	12.1	7.3

Age, body mass index (BMI), high-density lipoprotein cholesterol (HDL-C), non-HDL-C, total plasma cholesterol (TC), are given as mean (standard deviation); triglycerides (TG) values as median (range); and other values as number of individuals (n) with percentage (n/N) in parentheses.

*p < 0.05 versus control group; **p < 0.01 versus control group.

The present study had > 80% power to detect an association with ORs of ≥ 1.2 for alleles at 10% to 40% frequency for the total population and the subgroup analysis.

### *PDGFD *-858 variant was associated with risk of first-ever ICH

The distribution of *PDGFD *genotypes of -858 is shown in Table 2, and fulfilled expectations of Hardy-Weinberg equilibrium in both cases and controls. Genotype (CC-AC-AA) analyses were conducted for dominant model (AC+AA vs. CC), additive model (CC *vs*. AC *vs*. AA) and recessive model (CC+AC *vs*. AA). The additive genetic model assumes that there is a linear gradient in risk between the CC, AC and AA genotypes (CC genotype baseline). After adjustment for age, gender, BMI, smoking, HDL, non-HDL-C, TC, TG, blood pressure and fasting blood glucose with multivariate logistic regression analysis, the presence of the AA genotype of the -858 locus showed significantly increased risk of ICH (dominant model: odds ratio [OR] 1.29, 95% confidence interval [CI] 1.00-1.68, *P *= 0.05; additive model: OR 1.24, 95% CI 1.01-1.52, *P *= 0.04; recessive model: 1.44, 95% CI 0.92-2.25, *P *= 0.107) than wild-type genotype.

**Table 2:** Associations of *PDGFD *-858A/C genotype with stroke

	**Genotype, n (%)**				**Adjusted ORs**	**Adjusted**
**Groups**	**CC**	**AC**	**AA**	**Models**	**(95% CI)**	**P Value**
	
Controls (n = 1528)	868 (56.8)	564 (36.9)	96(6.3)		1.00	
Cases (n = 1484)	775 (52.2)	595 (40.1)	114(7.7)	Additive	1.15 (1.01-1.32)	0.04
				Dominant	1.18 (0.99-1.39)	0.06
				Recessive	1.25 (0.90-1.74)	0.18
Thrombosis (n = 654)	342 (52.3)	261 (39.9)	51 (7.8)	Additive	1.13 (0.95-1.35)	0.15
				Dominant	1.14 (0.91-1.42)	0.23
				Recessive	1.28 (0.85-1.93)	0.25
Lacunar (n = 419)	228 (54.4)	159(38.0)	32 (7.6)	Additive	1.05 (0.86-1.27)	0.63
				Dominant	1.03 (0.81-1.31)	0.82
				Recessive	1.19(0.75-1.90)	0.46
ICH (n = 411)	205 (49.9)	175 (42.6)	31 (7.5)	Additive	1.24 (1.01-1.52)	0.04
				Dominant	1.29 (1.00-1.68)	0.05
				Recessive	1.44 (0.92-2.25)	0.107

Adjusted OR was obtained on multivariate logistic regression after controlling for age, gender, BMI, smoking, HDL-C, non-HDL-C, TC, TG, fasting plasma glucose, and hypertension. Additive model: CC *vs*. AC *vs*.AA; Dominant model: CC*vs*. AC/AA; Recessive model: CC/AC *vs*. AA.

Of the 411 ICH cases recruited for this study, 314 were hypertensive (with hypertension history) ICH and 97 were non-hypertensive. Further analyses showed that -858AA genotype conferred about 2-fold increase in risk of non-hypertensive ICH (dominant model: OR 2.1, 95%CI 1.34-3.29, *P *= 0.001; additive model: OR 1.75, 95% CI 1.24-2.46, *P *= 0.001; recessive model: OR 1.77, 95%CI 0.79-3.95, *P *= 0.17) (Table 3). No association was observed between the *PDGFD *variant and hypertensive ICH and atherothrombotic stroke. After correction for multiple testing by Bonferroni procedure, the association of -858AA genotype with the risk of non-hypertensive ICH remained statistically significant.

**Table 3:** Associations of *PDGFD *-858A/C genotype with hypertensive and non-hypertensive ICH

	**Genotype, n (%)**				**Adjusted ORs**	**Adjusted**
**Groups**	**CC**	**AC**	**AA**	**Models**	**(95% CI)**	**P Value**
	
**Hypertensive ICH**						
Controls (n = 445)	239 (53.7)	176 (39.5)	30 (6.8)		1.00	
Cases (n = 314)	165 (52.5)	126 (40.1)	23 (7.4)	Additive	1.01 (0.78-1.31)	0.96
				Dominant	0.94 (0.68-1.31)	0.73
				Recessive	1.15 (0.62-2.15)	0.65
**Non-hypertensive ICH**						
Controls (n = 1083)	629 (58.1)	388 (35.8)	66 (6.1)		1.00	
Cases (n = 97)	39 (40.2)	50 (51.5)	8 (8.3)	Additive	1.75 (1.24-2.46)	0.001
				Dominant	2.10 (1.34-3.29)	0.001
				Recessive	1.77 (0.79-3.95)	0.17

Adjusted OR was obtained on multivariate logistic regression after controlling for age, gender, BMI, smoking, HDL-C, non-HDL-C, TC, TG, fasting plasma glucose, and hypertension. Additive model: CC *vs*. AC *vs*.AA; Dominant model: CC*vs*. AC/AA; Recessive model: CC/AC *vs*. AA.

### *PDGFD *-858 variant and risk of recurrent ICH

During a median 4.5 years (range, 0.1 to 5.4 years) of follow-up for 397 patients, a recurrent ICH occurred in 52 persons (13.1% of all index ICH), of which 39 were baseline hypertensive cases and 13 were baseline non-hypertensive. The risk time for recurrence in hypertensive and non-hypertensive ICH were shown in more detail **[**see Additional file 4**]**

**Figure 1:**
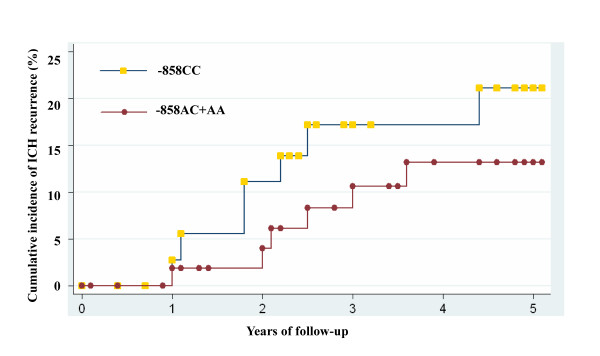
ICH Recurrence Rate in Patients with non-hypertensive First-ever ICH by *PDGFD *Variant -858A/C Genotypes*. *Kaplan-Meier curves for ICH recurrence by *PDGFD *-858A/C genotype among patients with non-hypertensive First-ever ICH. PDGFD = Platelet-derived growth factor D. ICH = intracerebral hemorrhage

Among patients with non-hypertensive ICH, the variant -858AA genotype was significantly associated with reduced risk of ICH recurrence (Figure 1), which was in contrast to its association with first-ever ICH. After adjusting for conventional cardiovascular risk factors, we found that -858AA genotype was associated with a reduced risk of ICH recurrence (dominant model: adjusted hazard ratio [HR] 0.09, 95%CI 0.01-0.74, P = 0.025; additive model: HR 0.21, 95% CI 0.04-1.16, P = 0.073; recessive model: HR 1.62, 95% CI 0.17-15.1, P = 0.671) in non-hypertensive patients. No association of *PDGFD *-858 variant with ICH recurrence was found in hypertensive patients.

### *PDGFD *promoter-activity analysis

**Figure 2:**
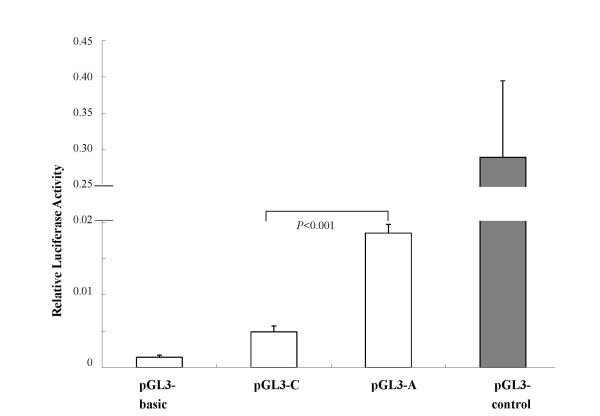
**SNP -858A-Bearing *PDGFD *Promoter Had Higher Transcription Activity**. The pGL3 luciferase reporter contained either the allele A (pGL3-A) or allele C (pGL3-C) at the promoter -858 locus. Values represent the average of 6 experiments and the bars represent the standard deviation. The pGL3-basic was used as a negative control without any promoter sequence, and pGL3-control as a positive control. PDGFD = Platelet-derived growth factor D.SNP = single nucleotide polymorphism; ICH = intracerebral hemorrhage

We investigated whether the polymorphism at position -858 affects the transcriptional activity of the *PDGFD *promoter in HCT-116 cells. The variant-858A-bearing PDGFD promoter exhibited a 3.78-fold increase in transcription activity than the variant-858C-bearing promoter. The pGL-3 empty vector had a negligible amount of the luciferase activity (Figure 2). The results supported that allele A has higher transcription activity than allele C.

## Discussion

The present study is the first clinical investigation of the significance of PDGF-D regarding the incidence and prognosis of ICH in a Chinese population. A common functional variation in the promoter region of *PDGFD *gene was found to be associated with more than 2-fold increase in risk of first-ever ICH in non-hypertensive cases, while reduced incidence of recurrent ICH.

In a small sample of south China, Han et al. have found that the G allele of rs7950273 of the PDGFD is associated with higher risk of ischemic stroke. However, the associations of PDGFD variants with other type of stroke, especially ICH have not been investigated [18]. In the present study, we found that the -858A/C variation in the *PDGFD *promoter region could lead to an enhancement of *PDGFD *promoter activity and consequently up-regulation of the expression of the gene. Interestingly, this functional variant was only associated with non-hypertensive cases, indicating that there are significant differences in genetic background, mechanisms and pathologies between the hypertensive and non-hypertensive ICH.

In this study, the relations between PDGFD variation and recurrent ICH were quite contrary to the contribution of the variant to the risk of first-ever ICH, which can probably be explained by the angiogenic and wound healing effects of PDGF-D. PDGF-D induces macrophage recruitment, extracellular matrix deposition, and blood vessel maturation, which are all part of process of angiogenesis and wound healing [15,19]. In addition, PDGFD is capable of inhibiting vascular leakage associated with growth factor-induced angiogenesis. This effect may be attributed to the PDGFD-induced stimulation of the proliferation and migration of SMCs, indicating that PDGFD is capable of stabilizing newly formed vessels through their effects on the SMCs [20].

Therefore, one can speculate that the enhancement of PDGF-D function is associated with proliferation and migration of SMCs, deposition of extracellular matrix, and degradation of vascular basement membrane, which on one hand lead to a loss of vessel wall integrity, resulting in increased susceptibility to primary non-hypertensive ICH. On the other hand, the above processes are also important procedures for angiogenesis and are essential for the development of neovessels during the restoration of vasculature system of injured brain after primary ICH [21,22].

The present study has limitations. First, although the sample size of this study is large, the relatively small number of each stroke subtype, especially the small sample size of non-hypertensive ICH, is a limitation of the present study; therefore further large scale studies focused on non-hypertensive ICH will be needed. Second, multiple testing of the genetic associations must be noted. After Bonferroni correction for multiple testing, the only association that maintains is the association with non-hypertensive ICH. Third, for CT/MRI readers, the intrareader variability was not calculated. However, the confirmed diagnosis of each case was based on results of at least two CT/MRI readers and one neurologist; disparities were resolved by discussion. The accuracy of the diagnosis was guaranteed by these methods.

In conclusion, our observations suggest that *PDGFD *-858A/C variant is probably associated with risk for non-hypertensive ICH. Further studies should be conducted to reveal the role of PDGF-D at various stages of ICH development--beneficial, or deleterious.

## Competing interests

The authors declare that they have no competing interests.

## Authors' contributions

BY carried out the molecular genetic studies, participated in the sequence alignment and drafted the manuscript. CJ participated in the sequence alignment and was involved in critically revising the manuscript for importand intellectual content. SK and WY participated in the design of the study and performed the statistical analysis. HR conceived of the study, and participated in its design and coordination. All authors read and approved the final manuscript.

## Supplementary Material

Additional file 1 **Genotyping of -858 Site**. Methods of genotyping and sequencing analysis of the -858 site of *PDGFD*. Click here for file

Additional file 2 **Luciferase reporter assays**. The detailed descriptions of the luciferase reporter assays. Click here for file

Additional file 3 **Proportions from each of the clinical sites**. The detailed descriptions of the proportions from each of the clinical sites for cases and controls. Click here for file

Additional file 4 **The risk time for recurrence in ICH**. The detailed descriptions of the risk time for recurrence in hypertensive and non-hypertensive ICH. Click here for file
