# Supplementary material for: A functional variant in promoter region of platelet-derived growth factor-D is probably associated with intracerebral hemorrhage
Source: J Neuroinflammation. 2012 Jan 30;9:26. doi: 10.1186/1742-2094-9-26 (PMC3307028; doi:10.1186/1742-2094-9-26)
Supplement: Additional file 1 — Genotyping of -858 Site. Methods of genotyping and sequencing analysis of the -858 site of PDGFD. [file 1742-2094-9-26-S1.PDF]

Additional file_1

Genotyping of -858 Site

There is a natural digestion site of Hinf I in the -858 fragment. The fragment containing variant -858 was amplified and a resultant 433bp sequence was obtained with the use of the following primers: 5’-CTGTGAAGGCAAGTGAGC-3’ and 5’-TTGGGTGAGTCAAGGGTA-3’. The resultant polymerase chain reaction (PCR) products were digested with Hinf I (New England Biolabs, Beverly, Mass), which yield 2 DNA fragments of 166 and 267bp for the A allele on 3% agarose gel and only 1 band for the C allele. Genotyping was performed without knowledge of the case or control status. Reproducibility of genotyping was confirmed by sequencing analysis in 100 randomly selected samples, with 100% agreement.
