# Supplementary material for: A functional variant in promoter region of platelet-derived growth factor-D is probably associated with intracerebral hemorrhage
Source: J Neuroinflammation. 2012 Jan 30;9:26. doi: 10.1186/1742-2094-9-26 (PMC3307028; doi:10.1186/1742-2094-9-26)
Supplement: Additional file 2 — Luciferase reporter assays. The detailed descriptions of the luciferase reporter assays. [file 1742-2094-9-26-S2.PDF]

Additional file_2

Luciferase reporter assays

To determine the *PDGFD* promoter activity, the *PDGFD* promoter encompassing -858 variant (from -1168 to +185) from patients carrying -858AA and -858CC genotypes was first cloned into the pGEM-T Easy vector (Promega, Madison, Wisconsin) with the forward primer: 5'-GAGCTAGCGAGAATCCCAAAAGCCTCAA-3' (contained *NheI* restriction site) and the reverse primer: 5'-CTCTCGAGGCGGGGGTTGCAGAAGTGT-3' (contained *XhoI* restriction site). The fragment containing the *PDGFD* promoter was released from the pGEM-T Easy vector by digesting the vector with *NheI* and *XhoI* restriction enzymes and was then subcloned into the multiple cloning sites (*NheI* and *XhoI*) of pGL3-basic vector (Promega). The pGL3 vector contains the cDNA encoding firefly luciferase. When it was fused with a promoter and transfected into mammalian cells, the construct can be used to analyze the inserted promoter activity. The vector containing the -858A/A genotype was designated as pGL3-A and the vector containing the -858C/C genotype as pGL3-C. The *PDGFD* promoter sequences in both vectors were confirmed by direct sequencing. Twenty-four hours before transfection, 2×10^5 HCT-116 cells were seeded in each well of a 6-well plate. On the day of transfection, each well was contransfected with 20μg of the pGL3 vector and 0.8μg of the pRL-CMV vector (Promega) using LIPOFECTAMINE 2000 (Invitrogen Corp., Carlsbad, California). The pRL-CMV vector, containing the CMV enhancer and early promoter elements to provide high-level expression of Renilla

luciferase, was used as internal control to normalize firefly luciferase expression. Forty-eight hours after transfection, the cells were lysed in passive lysis buffer (Promega). Cell lysate was added to the luciferase substrate (dual luciferase reporter system, Promega), and firefly and Renilla luciferase activity was measured with a luminometer (SIRIUS, Pforzheim, Germany).
