# Supplementary material for: A functional variant in promoter region of platelet-derived growth factor-D is probably associated with intracerebral hemorrhage
Source: J Neuroinflammation. 2012 Jan 30;9:26. doi: 10.1186/1742-2094-9-26 (PMC3307028; doi:10.1186/1742-2094-9-26)
Supplement: Additional file 3 — Proportions from each of the clinical sites. The detailed descriptions of the proportions from each of the clinical sites for cases and controls. [file 1742-2094-9-26-S3.PDF]

Additional file_3

Proportions from each of the clinical sites for cases and controls.

Clinical Sites	Control Subjects	Stroke Cases
Total	1528	1484
Wuhan Tongji, n (%)	250(16.36)	272(18.33)
Tian Jin, n (%)	128(8.38)	184(12.40)
Chong Qing, n (%)	262(17.15)	200(13.48)
Yanzhou, n (%)	213(13.94)	197(13.27)
Bei Jing, n (%)	170(11.13)	194(13.07)
Xi An, n (%)	298(19.50)	273(18.40)
Wuhan Xiehe, n (%)	207(13.55)	164(11.05)
