# Supplementary material for: A functional variant in promoter region of platelet-derived growth factor-D is probably associated with intracerebral hemorrhage
Source: J Neuroinflammation. 2012 Jan 30;9:26. doi: 10.1186/1742-2094-9-26 (PMC3307028; doi:10.1186/1742-2094-9-26)
Supplement: Additional file 4 — The risk time for recurrence in ICH. The detailed descriptions of the risk time for recurrence in hypertensive and non-hypertensive ICH. [file 1742-2094-9-26-S4.PDF]

Additional file_4

The risk time for recurrence in hypertensive and non-hypertensive ICH

Follow time(year)	Number of recurrence in hypertensive ICH	Number of recurrence in non-hypertensive ICH
0.1	1	0
0.2	0	0
0.3	1	0
0.4	2	0
0.5	1	0
0.6	0	0
0.7	3	0
0.8	1	0
0.9	1	0
1	4	2
1.1	1	1
1.2	1	0
1.3	0	0
1.4	0	0
1.6	1	0
1.7	1	0
1.8	1	2
1.9	0	0
2	2	1
2.1	1	1
2.2	0	1
2.3	1	0
2.4	0	0
2.5	1	2
2.6	5	0
2.7	0	0
2.8	1	0
2.9	1	0
3	1	1
3.1	1	0
3.2	0	0
3.3	0	0
3.4	1	0
3.5	1	0
3.6	2	1
3.7	1	0
3.8	0	0
3.9	0	0
